# High maternal mortality estimated by the sisterhood method in a rural area of Mali

**DOI:** 10.1186/1471-2393-11-56

**Published:** 2011-08-03

**Authors:** Ingvill Aa, Mari A Grove, Anita H Haugsjå, Sven G Hinderaker

**Affiliations:** 1 Medical Faculty, University of Bergen, 5020 Bergen, Norway; 2 Normisjon, P.b.7153, St.Olav plass, 0130 Oslo, Norway; 3 Centre for International Health, Årstadveien 21, University of Bergen, 5020 Bergen, Norway

**Keywords:** maternal mortality, rural, sisterhood method, Mali

## Abstract

**Background:**

Maternal mortality is high in Mali. Nevertheless, there are few studies on this topic from rural areas, and current estimates are mostly based on studies from urban settings. Our objective was to estimate the maternal mortality ratio in Kita, rural Mali.

**Methods:**

Using the "sisterhood method", we interviewed participants aged 15-50 years from 20 villages in Kita, Mali, and thereby created a retrospective cohort of their sisters in reproductive age. Based on population and fertility estimates, we calculated the lifetime risk of maternal death, and from that the estimated approximate maternal mortality ratio.

**Results:**

The 2,039 respondents reported 4,628 sisters who had reached reproductive age. Of these 4,628 sisters, almost a third (1,233; 27%) had died, and 429 (9%) had died during pregnancy or childbirth. This corresponded to a lifetime risk of maternal death of 20% and a maternal mortality ratio of 3,131 per 100,000 live births (95% confidence interval 2,967-3,296), with a time reference around 1999.

**Conclusions:**

We found a very high maternal mortality in rural Mali and this highlights the urgent need for obstetric services in the remote rural areas.

## Background

Many women on the African continent die during pregnancy and childbirth. Africa has 18 of the 20 countries with highest maternal mortality ratio (MMR) in the world. A recent study by Hogan et al. [1] reported the MMR of Mali in 2008 to be 670 per 100,000 live births (95% confidence interval (CI) 422-1017). Estimates for previous years suggested a slowly decreasing MMR from 1980 (1125; 95% CI 712-1688) to 1990 (831; 95% CI 525-1263) and to 2000 (807; 95% CI 513-1258). However, the low precision of the estimates and the overlapping confidence intervals imply non-significant changes between most of the MMR estimates, at least in the years since 1990.

Mali is one of the poorest countries in the world ranking as number 175 out of 178 in the world development index of the United Nations [2]. Vital registration is not available in Mali and data on maternal mortality are mainly based on reviews of institutional case fatality rates. These show a significant reduction in the risk of institutional maternal death in recent decades [3]. A national referral system in Mali was introduced to improve access to emergency obstetric care and a study from Kayes region showed that after its introduction the risk of institutional maternal mortality was halved in only 2 years [4].

In many African countries there is no vital registration and national maternal mortality is often estimated from various smaller studies of various designs. These studies are usually from facilities and populations in urban settings and hence there is a need for studies from rural settings. This is particularly important as most of the population of Mali resides in rural areas; the 2006 Demographic and Health Survey from Mali reported the population to be 73% rural and 27% urban [5]. However, many issues make a study on maternal mortality in rural Africa challenging; a large sample size is required for these (relatively) rare events, maternal death often occurs at home, and follow-up studies take time. The "sisterhood method" for estimating the MMR is therefore an ideal method for estimating MMR in such settings as it requires a smaller number of respondents compared with vital registration and cohort studies; the data collection for this method is retrospective, simple, quick, and based on information about maternal deaths among the sisters of the respondents [6,7]. The method cannot be used to assess trends, however.

Our objective was to estimate the MMR in a rural setting in western Mali by using the sisterhood method.

## Methods

A maternal death is "the death of a woman while pregnant or within 42 days of termination of the pregnancy, irrespective of the duration and site of the pregnancy, from any cause related to or aggravated by the pregnancy or its management" [8]. In situations where identification of the cause of death is difficult another index can be used, namely "pregnancy related deaths". Pregnancy related deaths have the same definition as maternal deaths except they include all causes of death. The latter is simpler to measure by the sisterhood method. It provides a good estimate of maternal mortality, although the inclusion of deaths due to non-pregnancy related causes (such as violence and car crashes) means that some overestimation is inevitable. The reproductive age for estimating maternal mortality using the sisterhood method includes the years between 15 and 50 years, but in the study area it was observed that young women sometimes got married at 13 years of age. We therefore used 13 years of age as the start of reproductive age. Even though in our study area many respondents did not know the exact age of their sisters they clearly understood the concept of "reproductive age". Since a *sister *in this community could be any woman of same age group, or any woman with whom the participant has some family relation, we were careful to focus very specifically on sisters born to the same mother.

Our study design was based on the "indirect sisterhood method" and this represents a retrospective cohort study [6]. The respondents were males and females 15-49 years of age and the inclusion criteria for reported sisters required that they had been born to the same mother as the respondent and had reached reproductive age (13 years). By interviewing people about their sisters, we created a cohort of women considered to be at risk of maternal death. Follow-up information for the identification of maternal deaths was collected from the respondents through the same interview process.

The western part of Mali is *Région de Kayes *and borders Senegal, Guinea and Mauritania. It is one of the poorer and less developed regions of Mali. The 2006 Demographic and Health Survey from Mali reported illiteracy to be 73% for females and 64% for males [5]. The infrastructure is not well developed, without proper roads and with limited access to health services, especially during the rainy season. It is estimated that in Kayes region as a whole 35% of the population need to access transport services in order to reach health facilities, and this is especially pronounced in rural areas [5]. However, more recently a new main road passable in all seasons has been constructed in the area. Deliveries often take place at home without a skilled birth attendant, particularly in rural areas [9].

The study setting was an area of 20 rural villages of *Cercle de Kita *in *Region de Kayes *of Mali. A development assistance project with emphasis on health issues was initiated here by expatriates in collaboration with national authorities as there was a need to improve knowledge about preventing maternal deaths in the area. Urbanization during the time in question has not been high [10]. This is important as migration could have compromised the completeness of the information gathered by failing to report dead sisters who had migrated. Sample size calculations were carried out to ensure a reasonably reliable estimate of maternal mortality. In order to estimate the MMR with 80% power and 95% confidence level, given a mortality level between 1,500 and 2,500 per 100,000 live births, at least 3000 sisters are needed.

The outcome measure was the maternal mortality ratio (MMR), where the numerator was the number of maternal deaths that occurred during a calendar year, and the denominator was the number of live births in the same year. The age of the respondents was 15-49 years and this was reflected in the "observation period" of the sisters at risk and in the MMR calculation. Thus, the estimated MMR did not refer to the MMR during the period of data collection; rather, the calculated MMR applied to the calendar period when the sisters in the study lived through their reproductive years. Analyses were disaggregated by the location of the respondents in order to obtain estimates of village-specific maternal deaths, i.e. respondent location was used as a proxy for the location of the sister. Age at death was not available in our study. The indirect sisterhood method of estimating MMR has been used and validated in previous studies [7,11].

### Data collection

Data collection was carried out during October 2007 by local health workers, supervised by some of the authors. The local health workers were fluent in the local language and did not get remunerated for this study. Respondents from the general population were approached at random in public places in 20 villages. In addition to recording the age of the respondent the questions posed were as follows: 1) How many sisters have you ever had who reached reproductive age (13 yrs)? 2) How many of these sisters are alive? 3) How many of these sisters are dead? 4) How many of the dead sisters died during pregnancy, labour or within 42 days after the delivery? These four questions were translated into French, and from French into the local language with the help of field assistants. The local health workers who carried out the interviews were trained in the interviewing process and their interview technique were pilot-tested on a few persons before data collection was initiated. The period of 42 days in question 4 was rounded to 6 weeks.

For each 5-year group the number of sisters exposed to the risk of maternal death and the duration of their exposure (i.e., the number of reproductive years) was calculated by multiplying the number of sisters by an empirical adjustment factor for that age group (e.g., a respondent above 60 years would have a factor of 1.0 meaning that all their sisters had been observed for their entire reproductive period). This adjustment factor was assumed to be independent of location. The lifetime risk of maternal death (LTR) was calculated using the total number of maternal deaths divided by the estimated total number of sisters exposed. Estimates of TFR were obtained from United Nations reports which estimated that the total fertility rate (TFR) in Mali decreased moderately by 16% from 1975-1980 to 2005-2010 [12]. In 1996, the TFR in Mali was 6.7; in Bamako it was 4.7 and in Kayes it was 6.9, which we used in our calculations [13]. The following formula from Graham [6] was used to calculate the approximate MMR from the LTR: MMR = 1 - [(1-LTR)1/TFR]. The formula for calculating the time reference was obtained from the same source. Ethics approval for research was obtained from *Centre National de la Recherche Scientifique et Technologique *in Bamako.

## Results

Of the 2,070 interviews carried out, 31 were excluded due to inconsistencies, errors or missing values. Among the remaining 2,039 participants there were 748 men (37%) and 1,291 women (63%), reporting a total of 4,628 sisters of reproductive age of whom 1,233 (27%) had died; 429 (9%) had died during pregnancy and childbirth. Table 1 shows the estimated number of sisters exposed, by 5-year age group, and the estimated lifetime risk for the entire cohort. The total lifetime risk of maternal death was 19.7%, and given a TFR of 6.9 in the region this corresponded to an estimated maternal mortality ratio of 3,131 per 100,000 live births (95% CI 2,967-3,296). Assuming a lower TFR of 5.0 yielded an MMR of 4,294 per 100,000 live births (95% CI 4,072-4,520), while a TFR of 7.5 resulted in an MMR of 2,884 per 100,000 live births (95% CI 2,733-3,036). The approximate time reference for the MMR estimate was the year 1999, eight years before the interviews. Village-specific analyses showed that more than 15% of the sisters identified from the villages of Makana, Bafing-Makana, Koba, Fangalakouta and Kologon had suffered a pregnancy related death (Table 2). In these same villages, approximately 40% or more of the sisters identified had died from any cause.

**Table 1:** Responses of 2039 respondents about their sisters' vital status and lifetime risk of maternal death in Kita, Mali, 2007.

Col.1	Col.2		Col.3	Col.4		Col.5		Col.6	Col.7	Col.8
**Age group of respondent (years)**	**No. of respondents (%)**		**No. of sisters ≥ 13 y**	**No. of sisters ≥ 13 y who died (%)**		**No. of maternal deaths (%)**		**Adjustment factor**	**Sisters exposed (Col3 × Col6)**	**Lifetime risk (Col5/Col7)**

15-19	485	(24%)	886	210	(24%)	81	(9%)	0.107	95	
20-24	294	(14%)	668	123	(18%)	38	(6%)	0.206	138	
25-29	248	(12%)	631	129	(20%)	50	(8%)	0.343	216	
30-34	288	(14%)	704	156	(22%)	49	(7%)	0.503	354	
35-39	250	(12%)	576	161	(28%)	57	(10%)	0.664	382	
40-44	237	(12%)	562	203	(36%)	77	(14%)	0.802	451	
45-49	238	(12%)	601	251	(42%)	77	(13%)	0.9	541	

**Total**	**2039**	**(100%)**	**4628**	**1233**	**(27%)**	**429**	**(9%)**		**2177**	**0.197**

**Table 2:** Reported vital status of sisters of 2039 respondents in 20 villages in Kita, Mali.

Village	Sisters who reached 13 y	**Sisters **≥ **13 y who died**		Maternal deaths among sisters ≥ 13 y	
Bafing-Makana^1^	217	127	(59%)	41	(19%)
Bagnakafata	297	70	(24%)	15	(5%)
Behon	210	41	(20%)	12	(6%)
Biliko	172	18	(10%)	6	(3%)
Bougaribaya	359	103	(29%)	31	(9%)
Darsalam	344	73	(21%)	11	(3%)
Fangalakouta^2^	214	81	(38%)	39	(18%)
Faramansonia	147	14	(10%)	4	(3%)
Katabantakota	213	20	(9%)	10	(5%)
Katakoto	201	22	(11%)	6	(3%)
Koba	149	65	(44%)	28	(19%)
Kobarounto	345	120	(35%)	29	(8%)
Kokofata	188	67	(36%)	23	(12%)
Kologon^3^	205	107	(52%)	37	(18%)
Koronouna	156	21	(13%)	6	(4%)
Kouloubou	401	56	(14%)	27	(7%)
Koumakire	210	63	(30%)	15	(7%)
Makana^4^	261	127	(49%)	74	(28%)
Sitaninkoto	339	38	(11%)	15	(4%)
Total	4628	1233	(27%)	429	(9%)

1. Bafing-Makana: 60 km from nearest health centre;

2. Fangalakouta: 50 km from nearest health centre;

3. Kologon: 25 km from nearest health centre;

4. Makana: 8 km from nearest health centre, isolated in rainy season

## Discussion

Our study from rural Mali documented a maternal mortality ratio (MMR) of more than 3,000 per 100,000 live births. This MMR is much higher than the latest national estimates suggesting the MMR in Mali to be below 1000 maternal deaths per 100,000 live births [1,14]. Our study appears to be the first study on MMR from rural Mali, and it estimated the MMR with a time-reference point around the calendar year 1999. Other studies show a lower MMR than ours in Mali, and this may be partly because our study area was distinctly rural, as opposed to the location of studies that informed other estimates. A population based prospective follow-up study with data from the early 1990s with 5,782 pregnant women in urban/semiurban Mali observed 15 deaths and yielded an MMR of 327 per 100,000 live births (95% CI 180-510) [15]. An ecological study with data mostly from the 1990s found, not surprisingly, that the MMR was higher in rural (601/100,000 live birth, 95% CI 529-679) than in urban areas (241/100,000 live births, 95% CI 172-330). This likely reflects the differential availability of emergency obstetric services [16].

Our study provides a quick reference point for MMR in a rural poorly developed area with a particularly low literacy rate and poor access to health services. Some villages had particularly high maternal mortality, and these villages also had a higher overall mortality rate. Villages with high mortality rates were all remotely located and this underscores the importance of access to health services for the prevention of maternal deaths. Efforts to improve the infrastructure and access to health services in rural areas will probably have a great impact on maternal health. Our study was not designed to detect recent changes in maternal mortality, however, and cannot provide estimates of changes in maternal mortality.

The youngest group of respondents in our study reported that 81 (9%) of their sisters suffered a maternal death. This represents a very high maternal mortality experience as the number of sisters exposed to maternal death was small (95). Age of marriage in the study area was as low as 13 years, in contrast to the 15 years observed in other studies [6]. Low age at marriage could potentially explain the high MMR among the sisters of the youngest respondents, but we could not verify this in our study as we did not have information on the age of the deceased. The deaths in question would have been more recent and information from the youngest respondents was likely more rather than less reliable. Excluding the youngest age group of respondents from the cohort of respondents did not alter the estimated MMR substantially; the MMR remained above 2500 deaths per 100,000 live births. Also, sensitivity analyses showed that alternative assumptions about fertility rates (TFR 9% higher or 28% lower), still resulted in MMR estimates that were well above 2,500 per 100,000 live births. Furthermore, migration out of this area may have led to respondents to miss recalling some of their dead sisters, hence underestimating the maternal mortality ratio.

Deaths due to abortions and ectopic pregnancies may have been misclassified as deaths due to causes unrelated to pregnancy or childbirth and this could have also resulted in an underestimate of the MMR. The frequency of ectopic pregnancies may be in the order of 1% of all pregnancies [17]. Although abortion rates have been increasing in this area the issue remains a sensitive topic and abortion related deaths may have been concealed by respondents. A few abortion related deaths were reported, although we suspect that such deaths may have resulted in an underestimated rather than overestimated MMR.

Our study has several limitations. It refers to the prevailing MMR around 8 years before data collection, and cannot provide the most recent MMR for the study area. Our study sample was taken from Kita and the situation is probably fairly similar to other remote rural areas of Mali. However, differences in access to health services could easily make our results less generalizable to other rural areas. The very high illiteracy rate could have led to misunderstandings during the interviews. To avoid this problem we selected data collectors who were permanent residents and health facility workers in the area; they knew the language and culture very well. Repeated interview for verification of the response was not done. However, looking at the data we see that on average the respondents had between 2 and 3 sisters, increasing by age of respondent. This is expected in a setting with a TFR around 6, and suggests that the study respondents' information was reliable.

Rural areas of Mali need continued strong support for emergency obstetric care services, combined with improvement in roads and transport. As previously mentioned, one important change in health services in the study area had been introduced [4], with the construction of a new main road. The national policies pertaining to emergency obstetric care have changed following WHO recommendations and this includes a system for referral and transport, and cost sharing with communities [18]. In the southern region of Mali, emergency obstetric care services have been organized with assistance from the Save the Children Fund. Although increased utilization of these health services has occurred, the services still need quality improvement [19].

Another limitation of our study is that we do not know where the sisters in the cohort were living, and we used the village of the respondents as a proxy for the residence of their sisters. It is not surprising that the maternal mortality varies between villages, as access to health services differs from one village to another. However, our sample size was not designed to permit village-specific comparisons. Nevertheless, our findings are plausible and show that more remote villages have more maternal deaths. The regional authorities should ensure that emergency obstetric care services are offered to every woman in need, taking into account the large seasonal differences in transport accessibility. Furthermore, creating trust by giving good quality services is important for increasing health service utilization [20].

## Conclusion

Our study showed that in this remote rural area of Mali there was an alarmingly high maternal estimated mortality ratio of 3,131 (95% CI 2967-3296) maternal deaths per 100,000 live births. The government's focus on emergency obstetric care services will hopefully improve the situation for mothers.

## List of abbreviations

CI: confidence interval; LTR: life time risk (of maternal death); MMR: maternal mortality ratio; TFR: total fertility rate.

## Competing interests

The authors declare that they have no competing interests.

## Authors' contributions

IA and MAG planned the study, collected, entered and analysed the data and wrote the first draft. SGH supervised the whole process, reviewed and modified the drafts. AHH assisted in the field, reviewed the drafts and participated in the discussion. All authors read and approved the final manuscript.

## Authors' information

The authors IA and MAG are medical undergraduates at University of Bergen. AHH is a public health nurse and was a missionary in Mali. SGH is a medical doctor and associate professor at the University of Bergen.

## Pre-publication history

The pre-publication history for this paper can be accessed here:

http://www.biomedcentral.com/1471-2393/11/56/prepub
